# Serumfree culture of the suspension cell line QB-Tn9-4s of the cabbage looper, *Trichoplusia ni* , is highly productive for virus replication and recombinant protein expression

**DOI:** 10.1093/jis/14.1.24

**Published:** 2014-01-01

**Authors:** Gui-Ling Zheng, Hong-Xu Zhou, Chang-You Li

**Affiliations:** Key Lab of Integrated Crop Pest Management of Shandong Province, College of Agronomy and Plant Protection, Qingdao Agricultural University, Qingdao Shandong 266109, China

**Keywords:** insect cell lines, population doubling time, virus production

## Abstract

Serumfree cultures of insect cells play an important role in the fields of protein engineering, medicine, and biology. In this paper, the suspension cell line QB-Tn9-4s of *Trichoplusia ni* (Hübner) (Lepidoptera: Noctuidae) was successfully adapted to serumfree Sf-900 III medium and passaged for 52 generations. The adapted QB-Tn9-4s cells grew faster. Their population doubling time shortened from 27.4 hr in serum-containing medium to 24.1 hr, and their maximal density increased by 1.83-fold, reaching 3.50 ×10 ^6^ cells/mL in serumfree culture in T-flasks. The cells readily adapted to spinner culture, with maximum cell density of 4.40 × 10 ^6^ cells/mL in a spinner flask. Although the infection rate of *Autographa californica* multiple nucleopolyhedrovirus and production of occlusion bodies (OBs) of the adapted QB-Tn9-4s cells were 91.0% and 85.4 OBs/cell, respectively, similar to those of QB-Tn9-4s cells cultured in serum-containing medium and control BTI-Tn5B1-4 cells, their budded virus titer was 4.97 ×10 ^7^ TCID50/mL, significantly higher than those of the latter two. In addition, the expression levels of β-galactosidase at six days postinfection and secreted alkaline phosphatase at seven days postinfection in the adapted QB-Tn9-4s cells reached 2.98 ± 0.15×10 ^4^ IU/mL and 3.34 ± 0.13 IU/mL, respectively, significantly higher than those of QB-Tn9-4s and control BTI-Tn5B1-4 cultured in serum-containing media. The above findings establish a foundation for industrial production of virus and recombinant proteins in QB-Tn9-4s serumfree culture.

## Introduction

Insect cell lines are of great importance in the production of baculovirus and recombinant proteins. They are generally cultured in media containing a certain percentage of serum to support cell growth and proliferation. However, serum is expensive and contains complex components detrimental to separation, purification, and detection of culture products, limiting the application of insect cells. Thus, developing serumfree cultures of insect cell lines is desirable in cell, genetic, and protein engineering, medical biology, biotechnology, and the production of baculovirus and recombinant proteins ( Agathos 2007 ; Hashimoto et al. 2010 ). A variety of insect cell lines have been successfully cultured in serumfree media ( Ikonomou et al. 2002 ; Lua et al. 2003 ; Imanishi et al. 2012 ). Among them, Sf-21 and its clonal isolate Sf-9 of the fall armyworm, *Spodoptera frugiperda* (Smith) (Lepidoptera: Noctuidae) and BTI-Tn5B1-4 (High Five) of the cabbage looper, *Trichoplusia ni* (Hübner) (Lepidoptera: Noctuidae), have been widely applied to virus production and recombinant protein expression and cultured in serumfree media ( Granados et al. 2007 ). Inlow et al. (1989) showed that Sf-9 had a shorter population doubling time in a serumfree suspension culture than in a serum-containing culture. Kwon et al. (2003) compared the growth and protein expression of Sf-9, Sf-21, and BTI-Tn5B1-4 in four different serumfree media and found that both Sf-9 and BTI-Tn5B1-4 cells possessed advantages and disad-vantages in actual application. BTI-Tn5B1-4 cells are highly susceptible to baculovirus and could provide superior production of occlusion bodies (OBs) and recombinant proteins when compared to other insect cell lines. On a per milliliter basis, BTI-Tn5B1-4 cells produce five-to seven-fold of heterolo-gous proteins compared with Sf-9 cells ( Wickham et al. 1992 ; Davis et al. 1993 ). However, an alphanodavirus named Tn5 cell line virus was identified during production of hepatitis E virus-like particles in BTI-Tn5B1-4 cells infected with a recombinant baculovirus vector ( Li et al. 2007 ), thus there is a serious risk of contamination when using virus-like particles to produce vaccines or recombinant proteins for therapeutic purposes in BTI-Tn5B1-4 cells ( Merten 2007 ). Although Sf-9 cells could yield more budded virus (BV), but they produce less OBs and recombinant proteins. In addition, both Sf-9 and BTI-Tn5B1-4 are adherent cells. QB-Tn9-4s is a suspension *T. ni* cell line established in our laboratory. It has comparable production levels of OBs and recombinant proteins to BTI-Tn5B1-4 cells and does not agglomerate at high density in culture ( Meng et al. 2008 ). In addition, QB-Tn9-4s cell line does not contain Tn5 cell line virus, thus it has application potentials in large-scale industrialized cultures ( Shan et al. 2011 ). Therefore, in this study, the QB-Tn9-4s cell line was adapted to a serumfree medium and tested for its biological characteristics. The results showed that in serumfree medium, QB-Tn9-4s cells could grow well and produce high levels of OBs and recombinant proteins, showing broad application potentials.

## Materials and Methods

### Materials and reagents

*T. ni* embryonic cell line BTI-Tn5B1-4 (High Five) ( Granados et al. 1994 ) and *S. frugiperda* ovarian cell line Sf-9 ( Pasumarthy and Murhammer 1994 ) were provided by Dr. Blissard, Boyce Thompson Institute of Cornell University. *T. ni* embryonic suspension cell line QB-Tn9-4s was established and preserved in our laboratory ( Meng et al. 2008 ).

*Autographa californica* multiple nucleopolyhedrovirus (AcMNPV-1A) ( Wood 1980 ) and its β-galactosidase expressing recombinant strain AcMNPV-β-gal ( Wickham et al. 1992 ) and secreted alkaline phosphatase (SEAP) expressing recombinant strain Ac-MNPV-SEAP ( Davis et al. 1992 ) were kindly provided by Dr. Granados of Cornell University. All of the viruses were amplified and titrated following the plague assay method described by Wood (1977) using Sf-9 cells.

TNM-FH insect medium was prepared by supplementing Grace medium (Invitrogen, www.lifetechnologies.com ) with 0.3% lac-talbumin hydrolyzate (BD, www.bd.com ), 0.3% yeast extract (BD) and 10% fetal calf serum (FBS) (Thermo Scientific, www.thermoscientific.com ). Serumfree Sf-900 III medium was from Invitrogen and serumfree EX-CELL 420 medium was from SAFC Biosciences (Sigma-Aldrich, www.sigmaaldrich.com ). O-nitrophenyl-β-D-galactopyranoside (ONPG) and p-nitrophenyl phosphate (PNPP) were from Sigma-Aldrich.

### Cell culture

Cells at logarithmic growth phase were prepared as a suspension by gently pipetting up and down in a sterile culture hood. After being diluted five-fold with fresh medium, 5 mL of suspension was inoculated into each 25 cm ^2^ flask (Corning, www.corning.com ) and cultured at 27ºC in an incubator. Cells were observed with an IX71 inverted phase contrast microscope (Olympus, www.olympus-global.com ) and passaged every four days.

### Adaptation and culture in serumfree medium

QB-Tn9-4s cells stably-grown in TNM-FH medium were passaged and consecutively cultured in Sf-900 or EX-CELL 420 medium containing 6%, 4%, 2%, and 1% FBS for three passages each and then in serumfree Sf-900 III or EX-CELL 420 medium. After 10 passages in serumfree medium, they were used to measure their biological characteristics and observe their morphology. One hundred randomly selected cells were observed and photographed under a microscope and their size was measured using a microscopic scale.

### Measurement of cell growth and cell viability

Cells at the logarithmic growth phase were counted using a hemacytometer and diluted with fresh medium to 2 × 10 ^5^ cells/mL. For the T-flask culture test of QB-Tn9-4s and BTI-Tn5B1-4 in different media, 5 mL of cells were inoculated in each 25 cm ^2^ culture flask and cultured at 27ºC. Every 24 hr, cells in three randomly selected flasks were harvested and counted. For the spinner flask suspension culture test of QB-Tn9-4s in serumfree medium Sf-900 III, 80 mL of cells (2 ×10 ^5^ cells/mL) were seeded into a 125 mL spinner flask (Corning). Three cultures were prepared. The spinner flasks were placed on a magnetic stir plate and cultured at 27ºC under stirring at 100 r/min. Each 1 mL of the suspended cells were removed every 24 hr. The cell density was determined using a hemacytometer (Freshney 2005). The cell growth curves were generated, and the population doubling times were determined as previously reported ( Hayflick 1973 ). The viability of cells was measured by trypan blue staining method (Freshney 2005). In brief, an equal volume of cell suspension and 0.4% trypan blue solution was mixed and transferred to the chambers of the hemacytometer, and all cells (stained and unstained) in the 1 mm ^2^ were counted. At least 500 cells were observed in the different squares for each culture. The cell viability was calculated using the following equation: cell viability (%) = viable cells (unstained) / total cells (stained and unstained) ×100.

### Determination of virus infection rate and OB production

1 × 10 ^5^ of QB-Tn9-4s and BTI-Tn5B1-4 cells in TNM-FH medium and QB-Tn9-4s cells in Sf-900III medium were separately seeded in each well of 24-well plates (Corning) in triplicate, infected with AcMNPV-1A BV at a multiplicity of infection of 10 as previously reported ( Meng et al. 2008 ), and incubated at 27°C. The cultures were collected at four days postinfection, and infected and uninfected cells were counted using a hemacytometer to determine the ratio of infection. Cells were then disrupted by sonication to release OBs. OB concentration was measured using a hemacytometer, and the average production of OBs per infected cell was calculated as the ratio of the number of total OBs produced to the number of cells infected, as reported previously ( Wang and Granados 1992 ).

### Determination of BV production

1 × 10 ^5^ of QB-Tn9-4s and BTI-Tn5B1-4 cells in TNM-FH medium and QB-Tn9-4s cells in Sf-900III medium were separately seeded in triplicate into a well of 24-well plates, and infected with AcMNPV-1A BV at a multiplicity of infection of 10, as previously reported ( Meng et al. 2008 ). Culture medium was collected at four days postinfection by centrifugation at 5000 r/min for 3 min and was used as the virus source to measure BV titer, i.e. TCID50 per mL, using Sf-9 cells and the limiting dilution method ( Reed and Muench 1938 ).

### Measurement of recombinant protein production

1 × 10 ^5^ of QB-Tn9-4s and BTI-Tn5B1-4 cells in TNM-FH medium and QB-Tn9-4s cells in Sf-900III medium were separately seeded in triplicate into a well of 24-well plates and infected with recombinant virus AcMNPV-β-gal or AcMNPV-SEAP at a multiplicity of infection of 10, as described by Meng et al. (2008) . Samples were collected at various days postinfection and keep at -20ºC.

The production of recombinant β-galactosidase and SEAP was measured as reported previously by Davis et al. (1992) and Zheng et al. (2005) . In brief, for examining the level of SEAP expression, samples were sonicated and then heated at 65ºC for 5 min. 2 µL of each sample was mixed with 200 µL of SEAP assay buffer (1.0 mol/L di-ethanolamine, 0.5 mmol/L MgCl2, 10 mmol/L homoarginine, pH9.8) in a 96-well microtiter plate (Corning) and incubated at 37ºC for 10 min. Following the addition of 20 µL of 120 mmol/L p-nitrophenyl phosphate, absorbance at 405 nm was recorded at 1 min intervals in an MRX microplate reader (DYNEX Technologies, www.dynextechnologies.com ). SEAP activity was calculated according to the formula IU/mL = (∆OD405/min) ×(0.222 mL) / (18.8 mL/µmol cm) ×(0.002 mL sample) ×(0.56cm path length).

To measure β-galactosidase levels, samples were sonicated for 10 sec and centrifuged for 2 min at 12,000 ×g to remove debris. 2 µL of the supernatants was mixed with 0.8 mL Z-buffer (60 mmol/L Na2HPO4, 40 mmol/L NaH2PO4, 10 mmol/L KCl, 1 mmol/L MgSO4, 50 mmol/L β-mercaptoethanol, pH 7.4), and incubated at 28ºC for 10 min. The reaction was initiated by addition of 0.2 mL of pre-warmed ONPG (4 mg/mL ONPG in Z-buffer). After 2 min of incubation at 28°C, the reaction was stopped by addition of 0.5 mL of 1 mol/L Na2CO3. β-galactosidase activity was measured by readings at OD 420 nm and calculated according to the formula IU/mL = (OD420 ×1.5 mL) / (0.0045 ×2 min incubation ×0.002 mL sample).

### Statistical analyses

The data were expressed as mean ± standard deviation. Differences in AcMNPV infection rate, BV titer, OB production, and protein expression were analyzed by oneway ANOVA and Duncan’s pairwise multiple comparison test using DPS data processing system ( Tang and Zhang 2013 ).

## Results

### Adaptation of QB-Tn9-4s cells to serumfree medium

QB-Tn9-4s cells cultured in TNM-FH medium and EX-CELL 420 medium containing 6%, 4%, and 2% FBS had a similar growth rate and morphology. However, when cultured in EX-CELL 420 medium containing 1% FBS, their morphologies changed significantly. Some cells clustered together and particles appeared nearby. When cultured in serumfree EX-CELL 420 medium, many cells underwent deformation. Their boundaries blurred and the number of dead cells increased. To improve cell adaptability, cells were re-cultured in EX-CELL 420 medium with 1% FBS. After being passaged for four generations, cells were in a better growth status. However, when cultured again in serumfree medium, cells began to deteriorate; they were broken and eventually died. After repeated tries, cells failed to grow in serumfree EX-CELL 420 medium.

**Figure 1.**
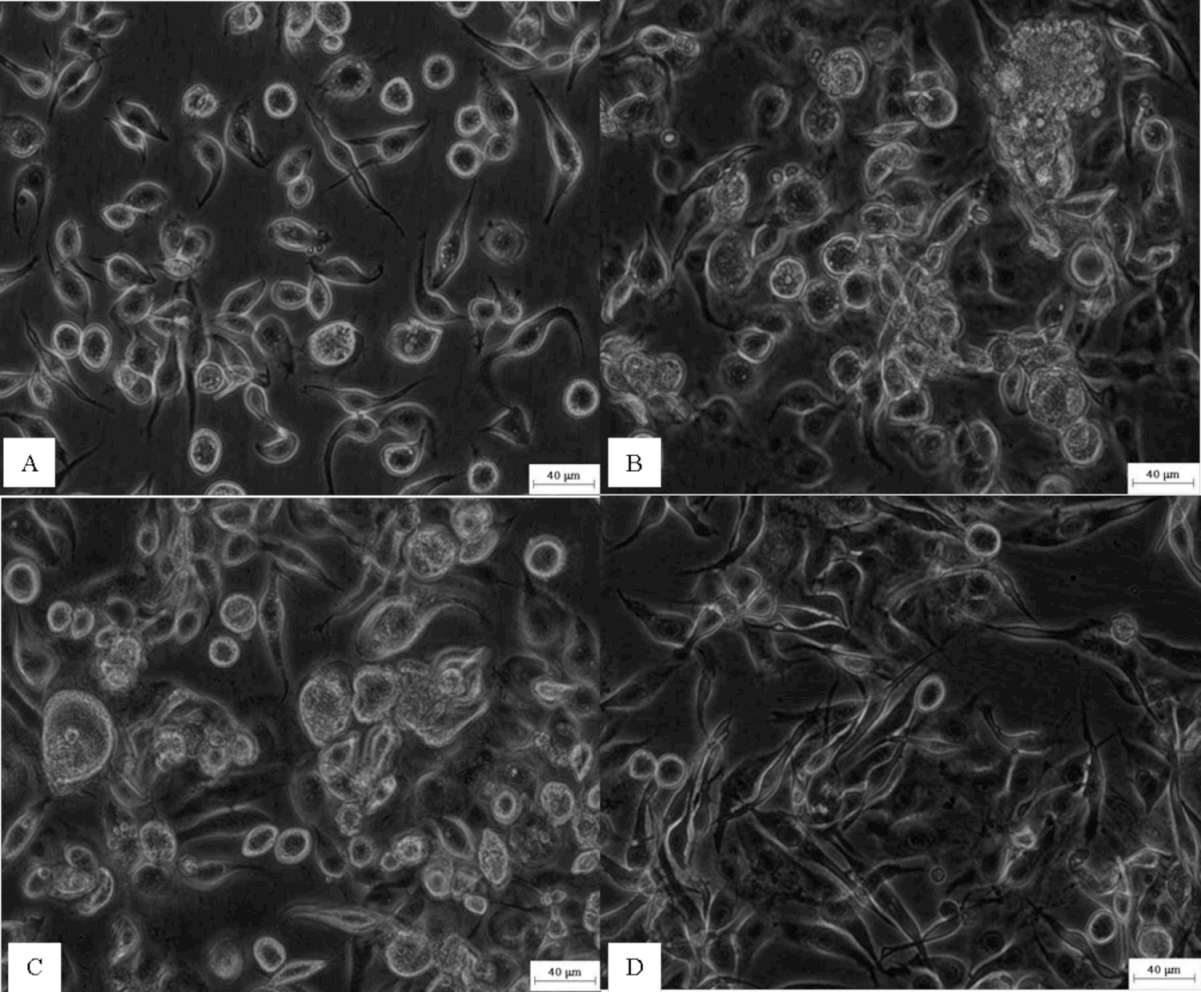
QB-Tn9-4s cells grown in serum-containing and serumfree media. A, QB-Tn9-4scells in TNM-FH medium. B, The early third generation QB-Tn9-4s cells in serumfree Sf-900 III medium. C, The late third generation QB-Tn9-4s cells in serumfree Sf-900 III medium. D, The fifteenth generation QB-Tn9-4s cells in serumfree Sf-900 III medium *.* Scale bar: 40 μm *.* High quality figures are available online.

QB-Tn9-4s cells cultured in TNM-FH medium ( Fig. 1A ) were well adapted to serumfree Sf-900 III medium compared to serumfree EX-CELL 420 medium. When serially passaged to Sf-900 III medium containing 6%, 4%, 2%, and 1% FBS, after three passages they were able to successfully passage at a 1:4 ratio (cell: medium). After passage to complete serumfree Sf-900 III medium, their growth became slower. Some cells underwent aggregation and deformation ( Fig. 1B ) and could not be normally passaged. After refreshing with 3 mL medium every four days, cells gradually grew normally ( Fig. 1C ) and passaged at a 1.5:3.5 ratio at confluency. About 50 days later, QB-Tn9-4s were able to grow stably in serumfree Sf-900 III medium and subcultured normally ( Fig. 1D ). Afterwards, cells grew significantly faster and passaged at a 0.8:4.2 ratio, indicating they were successfully adapted to serumfree medium. After passage 10, cells were used to measure their biological characteristics. So far, QB-Tn9-4s cells have been passaged to 52 generations in serumfree Sf-900 III medium and were able to successfully recover after three months of storage in liquid nitrogen.

### Cell morphology

The morphological characteristics of QB-Tn9-4s cells changed in the adaption process to the serumfree culture ( Fig. 1 ). The proportion of cells with a spindle shape increased from 70.1% in TNM-FH medium to 92.0% in serumfree medium, while that of cells with a round shape was reduced from 29.9% in TNM-FH medium to 8.0% in serumfree medium. In addition, the size of cells with a spindle shape increased from 57.7 ± 10.5 ×18.2 ± 2.1 µm in TNM-FH medium to 97.0 ± 17.0 ×19.1 ± 2.6 µm in serumfree medium, and the size of cells with a round shape decreased slightly from 22.2 ±3.0 µm in TNM-FH medium to 21.3 ± 3.1 µm in serumfree medium.

### The spinner culture of QB-Tn9-4s cells in serumfree medium

**Figure 2.**
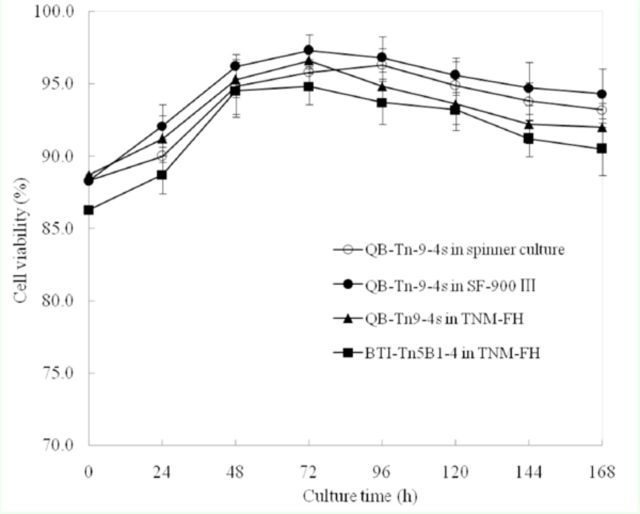
Viability of cells cultured in TNM-FH medium and serumfree Sf-900 III media. Data in the figure are expressed as mean ± SD. High quality figures are available online.

**Figure 3.**
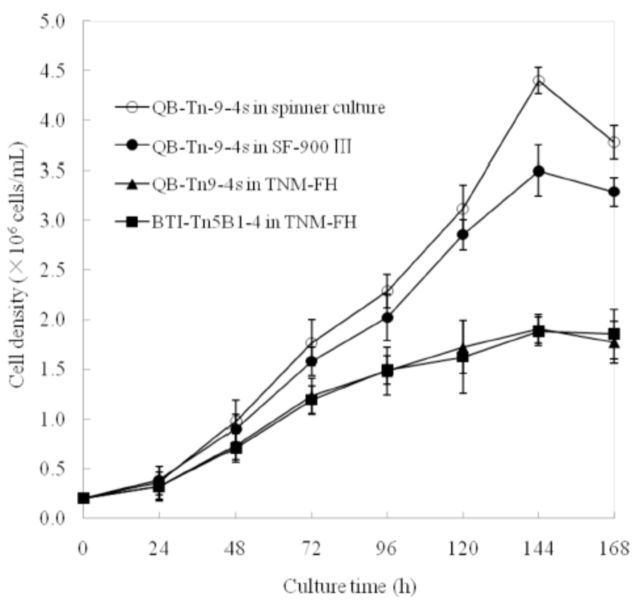
Growth curves of cells cultured in TNM-FH medium and serumfree Sf-900 III media. Data in the figure are expressed as mean ± SD. High quality figures are available online.

QB-Tn9-4s cells were readily adapted to spinner culture, and cell density increased generally during the early stage of suspension culture. In addition, cells were singles and not aggregated, and the cell mortality was less than 10% ( Fig. 2 ). The maximum cell density of QB-Tn9-4s was 4.40 ×10 ^6^ cells/mL in a spinner flask at 144 hr of culture ( Fig. 3 ). As cells grew beyond that time, some aggregation of cells was observed.

### Cell growth curve and population doubling time

The growth curves of QB-Tn9-4s cells in serum-containing and serumfree media in a T-flask and in a spinner flask were plotted using culture time as abscissa and cell density and cell viability as ordinate, respectively, and compared to that of control BTI-Tn5B1-4 cells. All cultures showed greater than 90% cell viability except BTI-Tn5B1-4 cells in TNM-FH at 24 hr after inoculation, which had only 88.7% cell viability ( Fig. 2 ). As shown in Fig. 3 , cell density increased significantly 24 hr after inoculation, indicating that the inoculated cells were less affected by mechanical injury and able to quickly adapt to the new culture environment. 144 hr after inoculation, the density of QB-Tn9-4s cells in serumfree Sf-900 III medium reached its peak of 4.40 ×10 ^6^ cells/mL in spinner flasks and 3.50×10 ^6^ /mL in the T-flask, respectively. The cell density (3.50 × 10 ^6^ /mL) was 1.83-fold of that in TNM-FH medium (1.91 × 10 ^6^ /mL) and 1.87-fold of that of control BTI-Tn5B1-4 cells in T-flask culture (1.88×10 ^6^ /mL). With the culture time increasing, nutrients in the medium were consumed and cellular metabolites were increased, resulting in slowly decreased cell growth rate, declined cell density, and an increased amount of aggregated cells.

The population doubling times of QB-Tn9-4s cells in serumfree medium in the spinner suspension and in the T-flask were 22.9 hr and 24.1 hr, respectively, both shorter than that of QB-Tn9-4s (27.4 hr) and of the control BTI-Tn5B1-4 cells (28.0 hr) in TNM-FH medium, indicating that QB-Tn9-4s grew faster in serumfree medium than in serum-containing medium and faster than the control BTI-Tn5B1-4 cells.

### Virus infection rate and yield

AcMNPV infection similarly changed the morphology of QB-Tn9-4s cells in TNM-FH medium and serumfree Sf-900 III medium. The cells grew slower and had condensated, aggregated chromatins; at three days postinfection, some cells appeared to have an enlarged nucleus and a small amount of OBs with strong refractivity in their nucleus; at four days postinfection, cells produced large amounts of OBs; at five days postinfection, large amounts of OBs were released into medium. Table 1 shows AcMNPV infection rate, OB yield, and BV titer in cells at different culture conditions. The infection rates of AcMNPV in QB-Tn9-4s cells cultured in both serumfree and serum-containing medium and control BTI-Tn5B1-4 cells in TNM-FH medium were greater than 90% with no significant difference. In addition, the average OB yields were higher than 83 OBs/cell. Although OB yields in QB-Tn9-4s cells cultured in serumfree and serum-containing media were higher than that of the control BTI-Tn5B1-4 cells, there were no significant differences.

**Table 1.**
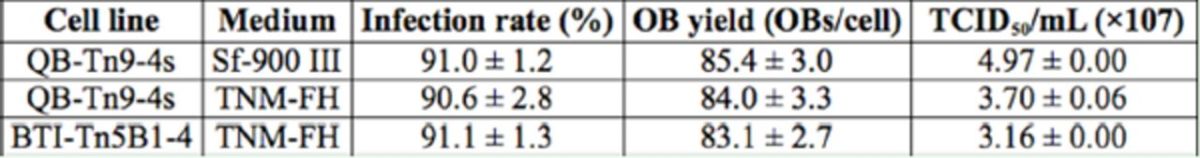
The infection rate, occlusion body (OB) yield, and budded virus titer of AcMNPV in different cell lines. Values in the table are mean ± SD.

The titer of BV was determined in triplicate in sf9 cells using culture supernatants collected at four days postinfection. As shown in Table 1 , the titer of BV produced by QB-Tn9-4s cells in serumfree Sf-900 III medium was the highest, followed by QB-Tn9-4s cells in TNM-FH medium, while that of BV produced by the control BTI-Tn5B1-4 cells in TNM-FH medium was the lowest. ANOVA showed extremely significant differences among the titers of BV produced at the three different conditions ( *P* < 0.01).

### Expression of recombinant β-galactosidase

**Figure 4.**
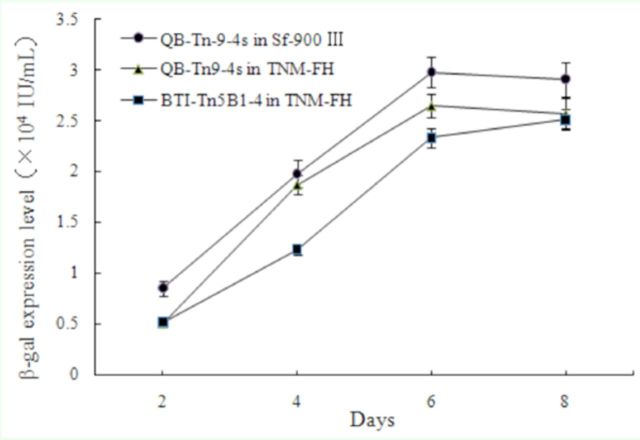
Beta-galactosidase expression level of cells cultured in different media at two to eight days postinfection. High quality figures are available online.

The expression levels of β-galactosidase at two, four, six, and eight days postinfection in cells infected with recombinant virus AcMNPV-β-gal are shown in Figure 4 . As shown, the expression level of β-galactosidase increased with the gradual increase of viral infection time, except that the highest level was seen six rather than eight days postinfection in QB-Tn9-4s cells cultured in serumfree SF-900 III medium. Moreover, at the same days postinfection, β-galactosidase expression level was the highest in QB-Tn9-4s cells cultured in serumfree SF-900 III medium, followed by QB-Tn9-4s cells cultured in TNM-FH medium, and the lowest was in control BTI-Tn5B1-4 cells. For example, at six days postinfection, β-galactosidase expression level reached a peak of 2.98 ± 0.15 ×10 ^4^ IU/mL in QB-Tn9-4s cells cultured in serumfree SF-900 III medium, followed by 2.65 ± 0.11 × 10 ^4^ IU/mL in QB-Tn9-4s cells in TNM-FH medium and 2.33 ± 0.09 × 10 ^4^ IU/mL in control BTI-Tn5B1-4 cells in TNM-FH medium. There were significant differences among β-galactosidase expression levels in these three conditions at six days postinfection ( *P* <0.05) based on Duncan’s multiple comparison test.

### Expression of recombinant secreted alkaline phosphatase (SEAP)

**Figure 5.**
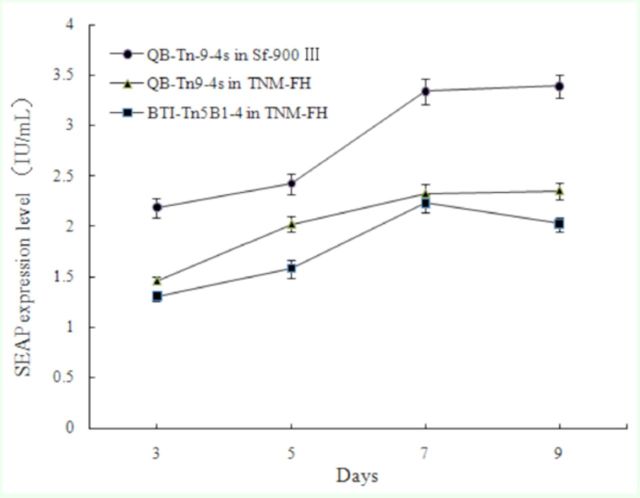
Secreted alkaline phosphatase expression level in cells cultured in different media at three to nine days postinfection. High quality figures are available online.

The expression levels of SEAP in cells infected with recombinant virus AcMNPV-SEAP at three, five, seven, and nine days postinfection are shown in Figure 5 . As shown, SEAP expression levels gradually increased with time in all cells except in the control BTI-Tn5B1-4 cells, which showed the highest SEAP expression at seven days postinfection rather than at nine days. At the same days postinfection, SEAP expression level in QB-Tn9-4s cells was higher when cultured in serumfree Sf-900 III medium than in TNM-FH medium. In addition, SEAP expression in QB-Tn9-4s cells was higher than in the control BTI-Tn5B1-4 cells. For example, at seven days postinfection, SEAP expression reached its peak level of 3.34 ± 0.13 IU/mL in QB-Tn9-4s cells in serumfree SF-900 III medium, which was extremely different from that of 2.32 ± 0.10 IU/mL in QB-Tn9-4s cells in TNM-FH medium and that of 2.23 ± 0.09 IU/mL in the control BTI-Tn5B1-4 cells at seven days postinfection ( *P* < 0.01) based on Duncan’s multiple comparison test. Moreover, SEAP expression in QB-Tn9-4s cells in TNM-FH medium was not significantly different from that in the control BTI-Tn5B1-4 cells at seven days postinfection ( *P* > 0.05).

## Discussion

With the rapid advancement of serumfree cell culture techniques in recent years, a variety of insect cell lines have been successfully cultured in corresponding serumfree media ( Ikonomou et al. 2002 ; Lua et al. 2003 ; Imanishi et al. 2012 ). However, the application of each medium is restricted to cell types because of their different adapt-abilities to various serumfree media. Kwon et al. (2003) showed that serumfree Sf-900 II medium was suitable for Sf-9 and Sf-21 cells, whereas serumfree Express Five medium was preferable for Tn5B1-4 cells. In this paper, QB-Tn9-4s cells were trained to survive in both serumfree EX-CELL 420 and Sf-900 III media. The results showed that they were able to grow in serumfree Sf-900 III medium, but not in EX-CELL 420 medium. Currently, QB-Tn9-4s cells have been passaged for 52 generations, and are still in good status, laying a foundation for further research and application of the cell lines.

In insect cell cultures, serum provides not only growth factors for cell growth and reproduction but also detoxifying and antioxidant proteins and protease inhibitors for cell protection ( Agathos 2007 ). In the adaption process of QB-Tn9-4s cells to serumfree medium, with reduction of serum concentration, cells were prone to aggregate, form many particles around areas with high cell density, and grow slowly. These phenomena may be due to the decreased protective effects in low serum medium and the mechanical damages caused by pipetting during the passaging process, which eventually led to poor cell morphology and aggregation growth. Renner et al. (1993) believed that damaged cells tend to aggregate because they could release a kind of DNA to bring cells together and thereby promote cell adhesion.

Researchers have studied and compared virus yield in a variety of insect cell lines in serumfree medium. Wang et al. (1992) showed that the productions of polyhedral and extracellular viruses of Sf-21, Sf-9, and Tn368 cells in serumfree medium were not significantly different from those produced in serum-containing medium. Chen et al. (1993) showed *T. ni* cells had higher Ac-MNPV titer and OB yield in serumfree EX-CELL 400 medium than in serum-containing TC199-MK medium, and OBs produced in serumfree culture were more virulent to *T. ni* larvae compared to those produced in serum-containing culture. In this study, the titer of AcMNPV BV produced in QB-Tn9-4s cells in serumfree Sf-900 III medium was significantly higher than that of BV produced in QB-Tn9-4s cells in TNM-FH medium and in the control BTI-Tn5B1-4 cells, indicating that QB-Tn9-4s cells in serumfree medium have application potential in studies on *in vitro* viral replication and proliferation.

In recent years, the insect baculovirus expression system has been widely used in theoretical research on proteins and massive production of genetically engineered vaccines and therapeutic proteins ( Granados et al. 2007 ; Smagghe et al. 2009 ). Thus, breeding insect cell lines with the ability to highly express recombinant proteins is of great importance in research and application of cell engineering and in serumfree culture systems for exogenous protein expression. Wang et al. (1992) measured the β-galactosidase expression level in Sf-21, Sf-9, and TN368 cells in serumfree medium and found that β-galactosidase expression level in serumfree EX-CELL 400 cultures of all the three insect cell lines was lower than that in serum-containing cultures. McKenna et al. (1998) measured the protein expression level in several *T. ni* embryonic cell lines in serumfree EX-CELL 400 medium and found that Tn-4B31 cells showed high protein expression levels, and the expression level of alkaline phosphatase was higher than that in BTI-Tn5B1-4 cells. Tatieek et al. (2001) compared the effect of different serumfree media on the expression of β-galactosidase in Sf-21 and BTI-Tn5B1-4 cells and found that β-galactosidase expression in Sf-21 cells was higher in EX-CELL 400 medium than in EX-CELL 401 and Sf900-II media, and expression of β-galactosidase in BTI-Tn5B1-4 cells was higher in EX-CELL 401 medium than in EX-CELL 405 medium. In this study, β-galactosidase and SEAP expressions in QB-Tn9-4s cells were higher in serumfree SF-900 III medium than in TNM-FH medium, indicating that protein expression of the same cell line was different in different media. Thus, it is feasible to select an appropriate serumfree medium for serumfree cultures of different insect cell lines to achieve high protein expression levels.

In this study, a new suspension cell line, QB-Tn9-4s, was successfully cultured in serumfree SF-900 III medium. The cells grew well in T-flasks and spinner flasks and had a high virus yield, titer, and recombinant protein expression level. These advantages provided broad prospects for development and in-depth research of the cell line. Further exploration on cell suspension culture in spinner flasks or shake-flasks and rational design of cell culture devices to study the metabolic characteristics of the cell line in large-scale cultivation and to optimize growth conditions would help to achieve large-scale industrialized cell cultures to express vaccines, enzymes, diagnostic reagents, and other important medical or valuable commercial goods.

## Abbreviations

AcMNPV: *Autographa californica* multiple nucleopolyhedrovirus
BV: budded virus
OB: occlusion body
SEAP: secreted alkaline phosphatase

## References

[R1] Agathos SN . 2007 . Development of serumfree media for lepidopteran insect cell lines. In: Murhammer DW, Editor . Methods in molecular biology, vol. 338: Baculovirus and insect cell expression protocols, 2/e . pp. 155 – 185 . Humana Press Inc . 10.1007/978-1-59745-457-5_8 17951770

[R2] Chen Q Mclntosh AH Yu Z Hong H Goodman CL Grasela JJ Ignoffo CM . 1993 . The replication of *Autographa californica* baculovirus (AcMNPV) in two lepidopteran cell lines grown in serumfree media . J. Invertebr. Pathol . 6 : 216 – 219 .

[R3] Davis TR Trotter KM Granados RR Wood HA . 1992 . Baculovirus expression of alkaline phosphatase as a reporter gene for evaluation of production, glycosylation and secretion . Bio/Technol. 10 : 1148 – 1150 . 10.1038/nbt1092-1148 1368794

[R4] Davis TR Wickham TJ McKenna KA Granados RR Shuler ML Wood HA . 1993 . Comparative recombinant protein production of eight insect cell lines . In Vitro Cell. Dev. Biol.-Anim. 29A ( 5 ): 388 – 390 . 8314732 10.1007/BF02633986

[R5] Granados RR Li GX Derksen ACG McKenna KA . 1994 . A new insect cell line from *Trichoplusia ni* (BTI-Tn-5B1–4) susceptible to *Trichoplusia ni* single enveloped nuclear polyhedrosis virus . J. Invertebr. Pathol . 64 : 260 – 266 .

[R6] Granados RR Li GX Blissard GW . 2007 . Insect cell culture and biotechnology . Virologica Sinica 22 : 83 – 93 .

[R7] Hayflick L . 1973 . Subculturing human diploid fibroblast cutures. In: Kruse PF, Patterson MK, Editors . Tissue culture methods and application . pp. 220 – 223 . Academic Press .

[R8] Hashimoto Y Zhang S Blissard GW . 2010 . Ao38, a new cell line from eggs of the black witch moth, *Ascalapha odorata* (Lepidoptera: Noctuidae), is permissive for AcMNPV infection and produces high leves of recombinant proteins . BMC Biotechnology 10 : 50 . doi: 10.1186/1472–6750-10–50 20602790 10.1186/1472-6750-10-50 PMC2906426

[R9] Ikonomou L Peeters-Joris C Schneider YJ Agathos SN . 2002 . Supernatant proteolytic activities of High-Five insect cells grown in serumfree culture . Biotechnol. Lett. 24 : 965 – 969 .

[R10] Imanishi S Kobayashi J Sekine T . 2012 . Serumfree culture of an embryonic cell line from *Bombyx mori* and reinforcement of susceptibility of a recombinant BmNPV by cooling . In Vitro Cell. Dev. Biol.-Anim. 48 : 137 – 142 . 22302376 10.1007/s11626-011-9465-9

[R11] Inlow D Shanger A Maioralla B . 1989 . Insect cell culture and baculovirus propagation in protein free medium . J. Tissue Culture Methods 12 : 13 – 16 .

[R12] Kwon MS Dojima T Park EY . 2003 . Comparative characterization of growth and recombinant protein production among three insect cell lines with four kinds of serum free media . Biotechnol. Bioproc. E. 8 : 142 – 146 .

[R13] Li TC Scotti PD Miyamura T Takeda N . 2007 . Latent infection of a new alphanodavirus in an insect cell line . J. Virol. 81 : 10890 – 10896 . 17686877 10.1128/JVI.00807-07 PMC2045576

[R14] Lua LHL Reid S . 2003 . Growth, viral production and metabolism of a *Helicoverpa zea* cell line in serumfree culture . Cytotechnology 42 : 109 – 120 . 19002933 10.1023/B:CYTO.0000015795.46813.44 PMC3449452

[R15] McKenna KA Hong HZ vanNanen E Granados RR . 1998 . Establishmant of new *Ttrichoplusia ni* cell line in serumfree medium for baculovirus and recombinant protein production . J. Invertebr. Pathol . 71 : 82 – 90 .

[R16] Meng MJ Li TL Li CY Li GX . 2008 . A suspended cell line from *Trichoplusia ni* (Lepidoptera): Characterization and expression of recombinant proteins . Insect Sci. 15 : 423 – 428 .

[R17] Merten OW . 2007 . Attention with virus contaminated cell lines . Cytotechnology 55 : 1 – 2 . 19002988 10.1007/s10616-007-9095-y PMC2289787

[R18] Pasumarthy MK Murhammer DW . 1994 . Clonal variation in the *Spodoptera frugiperda* IPLB-SF21-AE insect cell population . Biotechnol Prog . 10 : 314 – 319 . 7764939 10.1021/bp00027a012

[R19] Reed LJ Muench HA . 1938 . A simple method of estimation fifty percent endpoints . Am. J. Hyg . 27 : 93 – 497 .

[R20] Renner WA Jordan M Eppenberger HM Leist C . 1993 . Cell-cell adhesion and aggregation: Influence on the growth behavior of CHO cells . Biotechnol. Bioeng . 41 : 188 – 193 . 18609537 10.1002/bit.260410204

[R21] Shan M Zhang SY Jiang L Ma M Li GX . 2011 . Susceptibility to AcMNPV and expression of recombinant proteins by a novel cell clone derived from a *Trichoplusia ni* QAU-BTI-Tn9–4s cell line . Virologica Sinica 26 : 297 – 305 . 21979569 10.1007/s12250-011-3201-1 PMC8222455

[R22] Smagghe G Goodman CL Stanley D . 2009 . Insect cell culture and applications to research and pest management . In Vitro Cell. Dev. Biol.-Anim . 45 : 93 – 105 . 19247722 10.1007/s11626-009-9181-x

[R23] Tang QY Zhang CX . 2013 . Data Processing System (DPS) software with experimental design, statistical analysis and data mining developed for use in entomological research . Insect Sci . 20 : 254 – 260 . 23955865 10.1111/j.1744-7917.2012.01519.x

[R24] Tatieek RA Choi C Phan SE Palomares LA Sholer ML . 2001 . Comparison of growth and recombinant protein expression in two different insect cell lines in attached and suspension culture . Biotechnol. Prog . 17 : 676 – 684 . 11485429 10.1021/bp010061g

[R25] Wang P Granados RR . 1992 . Studies on serum free culture of insect cells for virus propagation and recombinant protein production . J. Invertebr. Pathol . 59 : 46 – 53 .

[R26] Wickham TJ Davis T Granados RR Shuler ML Wood HA . 1992 . Screening of insect cell lines for the production of recombinant proteins and infectious virus in the baculovirus expression system . Biotechnol. Prog. 8 ( 5 ): 391 – 396 . 1369220 10.1021/bp00017a003

[R27] Wood HA . 1977 . An agar overlay plaque assay method for *Autographa californica* nuclear polyhedrosis virus . J. lnvertebr. Pathol. 29 : 304 – 307 .

[R28] Wood HA . 1980 . *Autographa californica* nuclear polyhedrosis virus-induced proteins in tissue culture . Virology 102 : 21 – 27 . 18631644 10.1016/0042-6822(80)90066-5

[R29] Zheng GL Li CY Li GX Wang P Granados RR . 2005 . Construction and characteristics of a transformed lepidopteran cell clone expressing baculovirus *p35* . Chinese Sci. Bull. 50 : 2728 – 2732 .

